# Research progress on the role of IL-37 in gynecological and obstetrics-related diseases (Review)

**DOI:** 10.3892/ijmm.2026.5996

**Published:** 2026-09-21

**Authors:** Hongjing Wang, Renukha Arjune, Weiyu Gan, Na Guo, Nan Yu, Ting Xiong

**Affiliations:** 1 Department of Obstetrics and Gynecology, Tongji Hospital, Tongji Medical College, Huazhong University of Science and Technology, Wuhan, Hubei 430000, P.R. China; 2 Tongji Medical College, Huazhong University of Science and Technology, Wuhan, Hubei 430000, P.R. China

**Keywords:** interleukin-37, inflammation, endometriosis, polyendocrine metabolic ovarian syndrome, gynecological cancer

## Abstract

Interleukin 37 (IL-37), a member of the IL-1 family, is recognized as an anti-inflammatory cytokine. The anti-inflammatory properties of recombinant human (rh)IL-37 have been confirmed *in vitro* and its protective effects have been extensively demonstrated in various *in vivo* models. These include inflammatory disease models treated with rhIL-37 protein or transfected with plasmids encoding human IL-37 cDNA, as well as in IL-37 transgenic mice. IL-37 exerts its anti-inflammatory functions through intracellular pathways involving Smad3, and via extracellular signaling through the IL-18 receptor α chain (IL-18R)α/IL-1R8 complex or the IL-18 binding protein/IL-18Rβ axis. It maintains immune homeostasis by modulating the expression and activity of transcription factors, cytokines and chemokines, thereby balancing pro-inflammatory and anti-inflammatory responses. Beyond suppressing innate and adaptive immune responses, IL-37 alleviates gynecological conditions, inflammatory disorders and metabolic disturbances associated with polyendocrine metabolic ovarian syndrome, including obesity, insulin resistance and impaired glucose tolerance. Furthermore, IL-37 significantly inhibits the proliferation, invasion, metastasis and angiogenesis of ectopic endometrial cells and gynecological tumor cells. In recent years, interest in the role of IL-37 in gynecological and obstetric diseases has increased substantially. Accordingly, this review summarizes recent advances in understanding the involvement of IL-37 in inflammatory conditions and related tumors in gynecology and obstetrics. Unless otherwise specified, IL-37 in this article refers to the IL-37b isoform.

## Introduction

Interleukin (IL)-37 was first identified in 2000 and officially designated interleukin-IL-37 in 2010 (1). The IL-1 family comprises 11 members, most of which are pro-inflammatory cytokines (1). In contrast to its pro-inflammatory counterparts, IL-37 exhibits potent anti-inflammatory (2-4) and metabolic regulatory effects (5,6) in various inflammatory diseases, autoimmune disorders and cancers. The human IL-37 gene is located on chromosome 2, spans ~3.617 kb and contains six exons. Alternative splicing generates five distinct splice variants (IL-37a-e) (2). Among these, IL-37b, which comprises exons 1, 2, 4, 5 and 6, with only exon 3 absent and encodes 218 amino acids, is the longest and best-characterized isoform and is generally regarded as the principal biologically active form; IL-37a lacks exons 1 and 2, IL-37c lacks exons 3 and 4, IL-37d lacks exons 2 and 3 and IL-37e lacks exons 2-4 (2,7). Exons 1 and 2 of IL-37b encode an N-terminal pro-domain that contains a caspase-1 cleavage site, which is removed upon cytokine maturation. Exons 4-6 encode a canonical 12 β-strand trefoil structure characteristic of the IL-1 family cytokine domain, which mediates binding to the IL-18 receptor α chain (IL-18Rα) (8,9), suggesting that the IL-37a, b and d subtypes all have biological functions, while IL-37c and e do not. Despite extensive sequencing and database searches of the IL-1 gene locus, no murine homolog of IL-37 has been identified to date. Consequently, IL-37 transgenic (IL-37tg) mice have become an essential model for investigating the role of IL-37 in disease pathophysiology.

IL-37 exists in both monomeric and dimeric forms *in vivo*, with molecular weights of ~22 and 42 kDa, respectively (10,11). Native IL-37 forms a head-to-head homodimer that is considered autoinhibited (12), as dimerization restricts its interaction with IL-18Rα or IL-1R8. By contrast, the monomeric form exhibits significantly enhanced anti-inflammatory activity in various cell types (10,12,13). Based on these findings, it may be speculated that the paradox of elevated IL-37 alongside ongoing inflammation in certain inflammatory diseases may stem from partial homodimerization and self-inhibition of tissue-accumulated IL-37. Nevertheless, current studies merely quantify total IL-37 abundance, lacking sufficient evidence for its homodimerization and self-inhibitory effects in inflammatory diseases. In addition, the anti-inflammatory activity of IL-37 may also be regulated by microenvironmental factors. Can microenvironmental factors alter the balance between monomers and dimers? The above issues need to be experimentally verified.

The biological activity of IL-37 is regulated at multiple levels, including its production, receptor binding, proteolytic processing and signal transduction. A comprehensive understanding of these fundamental characteristics will enhance the current insight into the therapeutic potential of IL-37 in gynecological and obstetric diseases.

### Production of IL-37

IL-37 is constitutively expressed in various human cell types, including monocytes, activated B cells, natural killer (NK) cells, adipocytes and epithelial cells, and in tissues such as the thymus, lung, lymph node, breast, intestine, placenta and uterus (9). This basal expression is attributed to a conserved 10-nucleotide sequence element within the IL-37 coding sequence that partially overlaps with known or putative protein-binding sites involved in mRNA destabilization. These instability elements promote rapid degradation of IL-37-specific mRNA in unstimulated cells (14). Experimental evidence has shown that in stably transfected RAW264.7 mouse macrophage cells, no IL-37 protein expression was detected despite the presence of an active CMV promoter (9,14). A similar phenomenon was observed in IL-37 transgenic mouse models (15). However, IL-37 expression is closely associated with the inflammatory microenvironment and the presence of inflammatory cells (16). Upon inflammatory stimulation, IL-37 expression is markedly upregulated (2). For instance, lipopolysaccharide (LPS) stimulation strongly induces IL-37 expression in monocytes and macrophages *in vitro* (1). In addition to LPS, pro-inflammatory mediators such as IL-1β, IL-6, TNF-α and TGF-β1, have also been shown to effectively induce endogenous IL-37 expression in tissue cells (1). Notably, despite low constitutive expression, IL-37 levels are sufficient to exert potent immunoregulatory and anti-inflammatory effects. This represents a protective mechanism that limits excessive tissue damage and uncontrolled inflammation (11). Further studies have demonstrated that picomolar concentrations of IL-37 elicit robust anti-inflammatory responses in mice (17,18), whereas higher concentrations promote dimerization and autoinhibition, thereby attenuating its biological activity (10,13). In various gynecological and obstetric diseases as well as gynecological tumors, IL-37 expression levels vary, being either upregulated or downregulated (Table I). For instance, IL-37 levels are elevated in endometriosis, HIV infection and non-metastatic breast cancer, whereas they are decreased in adenomyosis, polyendocrine metabolic ovarian syndrome and endometrial carcinoma (Table I), suggesting context-dependent regulatory roles.

### Receptors of IL-37

Similar to Toll-like receptors (TLRs), members of the IL-1 receptor (IL-1R) family are structurally characterized by extracellular immunoglobulin-like domains and an intracellular Toll-IL-1 receptor (TIR) domain (19). The canonical signaling mechanism involves an IL-1 family ligand binding to a primary IL-1 receptor chain, which subsequently recruits a second IL-1 receptor chain (co-receptor chain) to initiate signal transduction (19). For instance, IL-37 belongs to the IL-1 ligand family, whereas IL-18Rα (also known as IL-1R5) and IL-1R8 are members of the IL-1R family (20). The anti-inflammatory activity of IL-37 requires simultaneous engagement of both receptors: IL-18Rα serves as the ligand-binding chain for IL-37, while IL-1R8 functions as the co-receptor (21,22). IL-1R8 (also termed TIR8 or SIGIRR) is a cell-membrane receptor that is essential for the signal transduction of extracellular IL-37 (23,24). Upon proteolytic cleavage, this receptor yields soluble IL-1R8 (sIL-1R8), which functions as an IL-37 antagonist. It blocks IL-37 binding to membrane-bound receptor complexes, thereby attenuating IL-37-mediated immune regulation and anti-inflammatory activities.

IL-18 binding protein (IL-18BP) is a high-affinity decoy receptor for IL-18 and functions as a natural antagonist of IL-18-mediated signaling (25). Notably, IL-18BP can bind not only to IL-18 but also to the anti-inflammatory cytokine IL-37 (26,27). Structurally, IL-37 shares two conserved amino acid residues with IL-18: Glu-35 and Lys-124 in IL-37 correspond to Glu-35 and Lys-89 in IL-18, respectively. These residues are critical for the interaction between IL-37 and IL-18BP (2,27). IL-37 binds to IL-18BP through these conserved sites, similar to IL-18, and subsequently associates with IL-18Rβ to form an IL-18BP-IL-37-IL-18Rβ ternary complex. This interaction prevents IL-18Rβ from participating in IL-18 signaling, thereby exerting an inhibitory effect (27-29).

Small mother against decapentaplegic (SMAD) family 3 (SMAD3) is a member of the SMAD family of intracellular signal transducer proteins (30). In the canonical SMAD signaling pathway, phosphorylated Smad2 and Smad3 associate with Smad4 to form a trimeric complex (Smad2/3/4), which translocates to the nucleus to regulate gene expression. Smad2 and Smad3 can also form functional dimers with Smad4 (Smad2/4 and Smad3/4) (31). Notably, although IL-37 possesses only a weak nuclear localization sequence, it can compete with Smad2 or Smad4 for binding to Smad3, thereby facilitating nuclear translocation of the IL-37/Smad3 complex (31). This complex promotes the production of anti-inflammatory factors and alleviates inflammatory responses (32), while simultaneously inhibiting multiple signaling pathways and the metastatic phenotype of tumor cells (31). In addition, a report have shown that intracellular IL-37 interferes with the TGF-β1 signaling cascade by interacting with Smad3, thereby attenuating liver inflammation and fibrosis (33). Another research group found that intervention with Smad3-specific small interfering RNA significantly weakened the inhibitory effect of IL-37 on IL-17, indicating that the TGF-β1-Smad3 pathway is involved in mediating the inhibitory action of IL-37 on IL-17 (1). Although the above studies have demonstrated that IL-37 can reduce the overall output of the TGF-β1 pathway, they have not provided direct experimental evidence that inhibition of the SMAD2/SMAD3/SMAD4 complex mediates IL-37-induced suppression of TGF-β1 signaling. Therefore, a direct causal link between Smad2/3/4 complex inhibition and IL-37-mediated suppression of TGF-β1 signaling remains to be experimentally established, and this represents an important direction for future research.

A 2010 study published in Nature Immunology (1) performed immunofluorescence staining with phosphorylated Smad3 antibodies and immunoprecipitation assays with non-phosphorylated Smad3 antibodies. The authors verified that IL-37b is capable of binding simultaneously to both phosphorylated and non-phosphorylated Smad3 in A549 and THP-1 cells. Consistent with these findings, Luo *et al* (31) further validated the interaction between IL-37 and Smad3 via immunoprecipitation experiments using non-phosphorylated Smad3 antibodies. Multiple review studies have also clarified that the binding of IL-37 to intracellular phosphorylated Smad3 promotes the nuclear translocation of IL-37 (4,11,32). These existing findings support two plausible mechanistic hypotheses: First, mature IL-37 may bind to non-phosphorylated Smad3 in the cytoplasm, thereby suppressing Smad3 activation and subsequent nuclear translocation. Second, IL-37 may interact with phosphorylated Smad3 to translocate into the nucleus and inhibit the transcription of downstream target genes. Nevertheless, these hypotheses remain to be biochemically validated. Specifically, phosphorylation-specific Smad3 antibodies are required to precisely identify the exact binding form of Smad3 that interacts with mature IL-37 in cells.

### Molecular processing and signal transduction of IL-37

Caspase-1 is a key protease required for the nuclear translocation of IL-37. The caspase-1 cleavage site is located between Asp20 and Glu21 within exon 1 of IL-37 (2,34). Newly synthesized IL-37 is produced as a precursor peptide, which is subsequently cleaved by caspase-1 in the cytoplasm to generate the mature form (34). Both the precursor and mature forms of IL-37 are biologically active and can be released from cells via an unconventional secretory mechanism (35).

**Figure 1:**
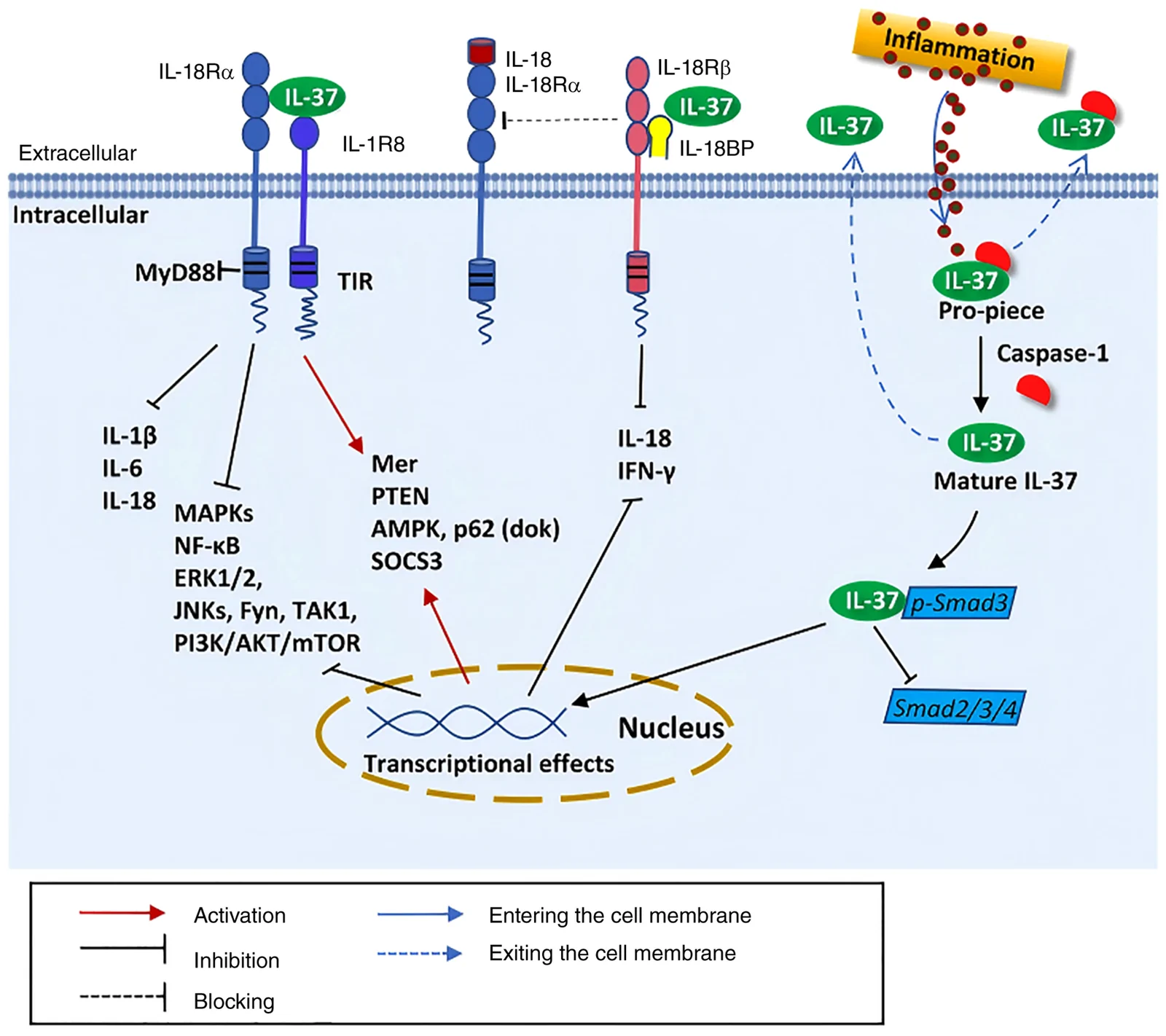
IL-37 inhibits inflammatory responses mainly through three potential pathways. Inflammatory stimulation upregulates the expression of IL-37. When extracellular IL-37 binds to the IL-18Rα/IL-1R8 complex, pro-inflammatory pathways are inhibited while anti-inflammatory pathways are activated. IL-1R8 plays a crucial role in this process, and its absence weakens the anti-inflammatory effects of IL-37. When extracellular IL-37 binds to IL-18BP, the β-chain of the IL-18R is subsequently recruited to form an IL-37-IL-18BP-IL-18Rβ ternary complex. This complex sequesters the β-chain, preventing it from forming a functional receptor complex with IL-18Rα, thereby inhibiting IL-18 activity. Intracellularly, caspase-1 cleaves the IL-37 precursor, and its mature form binds to phosphorylated Smad3, preventing the formation of the Smad2/3/4 complex. Subsequently, the IL-37/p-Smad3 complex translocates to the nucleus, where it regulates gene expression, enhances the production of anti-inflammatory factors and reduces inflammatory responses. IL-1β, interleukin-1β; IFN-γ, interferon γ; IL-18Rα, interleukin-18 receptor α chain; IL-18Rβ, interleukin-18 receptor β chain; IL-18BP, IL-18 binding protein; IL-1R8, interleukin-1 receptor 8; MyD88, myeloid differentiation primary response 88; TIR, Toll/interleukin-1 receptor; MAPKs, mitogen-activated protein kinases; NF-κB, nuclear factor κB; ERK1/2, extracellular signal-regulated kinase 1/2; JNKs, c-Jun N-terminal kinases; Fyn, Fyn tyrosine kinase; TAK1, TGF-β-activated kinase 1; PI3K, phosphoinositide 3-kinase; AKT, protein kinase B; mTOR, mammalian target of rapamycin; Mer, Mer tyrosine kinase; PTEN, phosphatase and tensin homolog; AMPK, AMP-activated protein kinase; p62(dok), downstream of kinase 1 (Dok1); SOCS3, suppressor of cytokine signaling 3; Smad3, SMAD family member 3; p-Smad3, phosphorylated Smad3.

IL-37 suppresses inflammation and modulates cellular function through three distinct pathways: Two extracellular and one intracellular (Fig. 1) (4,19,28). The first pathway involves IL-37 binding to the IL-18Rα/IL-1R8 receptor complex. On the cell surface, IL-37 binds to IL-18Rα and subsequently recruits IL-1R8 to form a tripartite complex. This assembly prevents the recruitment of the proximal MyD88 innate immune signal transduction adaptor, which is essential for signal transduction by IL-1, IL-18 and all TLRs. Consequently, downstream signaling cascades, including the MAPK, NF-κB and JNK pathways, as well as Fyn tyrosine protein kinase and TGF-β-activated kinase 1 activation, are inhibited. Simultaneously, this pathway activates anti-inflammatory mediators such as Mer tyrosine kinase and phosphatase and tensin homolog (PTEN), and induces p62 to further suppress inflammatory responses (19). Additionally, IL-37 promotes fatty acid oxidation and attenuates obesity progression by activating the AMPK signaling pathway (5). By attenuating the activation of ERK1/2, JNK and IKKα/β-kinases that promote insulin resistance by inhibiting insulin signaling-IL-37 enhances insulin sensitivity (5). IL-37 has also been shown to inhibit the growth of endometriotic lesions via the Akt and ERK1/2 signaling pathways, whereas IL-37 silencing produces the opposite effect (36,37). Studies in IL-1R8-deficient mice demonstrate that IL-37 fails to inhibit inflammasome activation, underscoring the essential role of IL-1R8 signaling in mediating the effects of IL-37 (19,37).

The second pathway involves IL-37 binding to IL-18BP and subsequently recruiting IL-18Rβ to form an IL-37-IL-18BP-IL-18Rβ ternary complex (4,20,27,28). IL-18BP is present in the extracellular space, where it binds to soluble mature IL-18, thereby preventing IL-18 from engaging its receptors and effectively neutralizing IL-18 activity (11,26). Notably, IL-37 binding to IL-18BP does not impair IL-18BP-mediated inhibition of IL-18; rather, it potentiates this inhibitory effect (20). In human NK cell lines, IL-37 enhances IL-18BP-mediated neutralization of IL-18 activity by 25-30%, thereby downregulating IL-18-induced IFN-γ production (26). The formation of the IL-37-IL-18BP-IL-18Rβ complex sequesters IL-18Rβ, preventing its incorporation into the IL-18-IL-18Rα-IL-18Rβ trimeric signaling complex. This effectively blocks IL-18 signal transduction and facilitates the anti-inflammatory actions of IL-37 (27).

The third pathway entails the binding of mature IL-37 to Smad3 in the cytoplasm (4,28,32). The C-terminal domain of mature IL-37 interacts with Smad3 and phosphorylated Smad3 (p-Smad3) facilitates the nuclear translocation of IL-37. Once in the nucleus, the IL-37/p-Smad3 complex suppresses multiple downstream signaling pathways, including ERK, JNK, p38 MAPK, PI3K, NF-κB and STAT3, thereby inhibiting inflammatory responses and exerting anti-tumor effects (31). IL-37 attenuates inflammation and mitigates sepsis-induced lung injury by modulating TGF-β and Smad3 signaling (38). Importantly, the D20A mutation (replacement of Asp20 with Ala) renders IL-37 resistant to caspase-1 cleavage, abolishes Smad3 binding and prevents nuclear translocation (39). Similarly, pharmacological inhibition of caspase-1 in IL-37tg mice impairs IL-37 function (39). Recent findings demonstrate that peritoneal macrophages from IL-37tg mice exhibit a 40-50% reduction in LPS-induced inflammatory cytokine production (IL-1β, IL-6, TNF-α and IFN-γ), whereas macrophages from IL-37-D20A-tg transgenic mice fail to exhibit this inhibitory effect (39). Furthermore, IL-37tg mice with Smad3 deficiency exhibit markedly attenuated anti-inflammatory responses following LPS stimulation compared to controls (2). Collectively, these findings confirm the critical role of Smad3-mediated nuclear translocation in the anti-inflammatory activity of IL-37.

In summary, IL-37 modulates the expression, phosphorylation and dephosphorylation of key signaling molecules through three distinct pathways, leading to significant alterations in fundamental biological processes, including cell proliferation, differentiation, angiogenesis, metabolism and inflammation.

## 2. IL-37 and gynecological diseases

### Role of IL-37 in endometriosis

Endometriosis is a chronic inflammatory gynecological disorder characterized by the presence, growth and infiltration of endometrial tissue (glands and stroma) outside the uterine cavity (40). This section focuses specifically on endometriosis occurring outside the uterine cavity; adenomyosis, characterized by the presence of endometrial tissue within the myometrium, will be discussed separately in the following section. The disease commonly manifests as chronic pelvic pain, dysmenorrhea, dyspareunia and menorrhagia (40). Although not life-threatening, these symptoms can severely compromise quality of life and daily functioning, imposing a substantial burden on both affected individuals, their families and society. Endometriosis is also associated with adverse pregnancy outcomes, including infertility, ectopic pregnancy, miscarriage, preterm labor, preeclampsia and intrauterine growth restriction (41). While the pathogenesis of endometriosis remains incompletely understood, accumulating evidence highlights inflammation and immune dysfunction as key contributors to its development.

In 2016, Jiang *et al* (42) first reported the involvement of IL-37 in the inflammatory process of endometriosis. The study demonstrated that IL-37 mRNA and protein expression levels were significantly elevated in the eutopic endometrium of women with ovarian endometriosis compared with control endometrium, with the highest expression observed in ectopic endometrium (42). These findings support the concept that IL-37 mediates a negative feedback mechanism to restrain excessive inflammation. The following year, Kaabachi *et al* (43) reported significantly higher IL-37 mRNA and protein levels in the peritoneal cavity and serum of patients with endometriosis, with expression levels correlating with disease severity. Furthermore, they observed a negative correlation between IL-37 mRNA expression and NF-κB mRNA expression in the peritoneal cavity of these patients (43), suggesting that endometriosis involves not only localized immune dysregulation but also a broader pattern of systemic inflammation.

Previous studies have shown increased numbers of activated immune cells in the peritoneal cavity and peripheral blood of patients with endometriosis, accompanied by abnormally elevated secretion of various inflammatory mediators (44,45). Among these, macrophages play a particularly important role in disease progression. IL-37, as a broad-spectrum anti-inflammatory cytokine, inhibits macrophage migration, proliferation and pro-inflammatory mediator production, while modulating apoptosis (36,46). It also reduces neutrophil activation and chemotaxis while enhancing NK-cell recruitment and infiltration (36,47,48). IL-37 can increase NK-cell cytotoxic activity in the presence of IL-15 (49). Mechanistically, IL-37 modulates the NF-κB pathway in M1-polarized macrophages, thereby suppressing pro-inflammatory cytokine expression (50). Intraperitoneal administration of rhIL-37 significantly reduced the levels of inflammation-related factors secreted by activated immune cells, including IL-1β, TNF-α, IL-6, IL-10 and TGF-β (36,37). *In vitro*, rhIL-37 treatment markedly decreased the expression of inflammatory factors in uterine and lesion tissue explants, as well as in human or murine endometrial stromal cells. Similar anti-inflammatory effects were observed in RL95-2 human endometrial cancer cells following rhIL-37 treatment (36). Of note, the above *in vivo* and *in vitro* experiments have demonstrated that IL-37 can significantly reduce the expression of IL-10 (36,37), whereas other studies report that IL-37 can upregulate IL-10 expression to alleviate myocardial injury caused by ischemia/reperfusion (48). This completely different result may be related to factors such as different cell types and experimental conditions.

The incidence of allergic diseases is increased in patients with endometriosis (51). Studies have reported elevated TNF-α levels, decreased IFN-γ and increased IL-4, IL-10 and IL-13 levels in these patients. These findings suggest a shift in cytokine balance from Type 1 T-helper cell (Th1) toward Th2 responses, resulting in immunosuppression that impairs effective clearance of ectopic endometrial tissue and thereby promotes disease progression (51,52). IL-37 has been shown to attenuate the expression of TNF-α, IL-4, IL-10 and IL-13, restore Th1/Th2 balance and ameliorate the immune microenvironment (52).

Dendritic cells (DCs) also play an undeniable role in endometriosis. Compared with controls, women with endometriosis exhibit a markedly reduced density of mature DCs and a significantly increased density of immature DCs (45,53), the latter of which contribute to endometriosis pathogenesis.

IL-37 exhibits significant context-dependent regulatory effects on DCs. In one study (52), CD4+ T cells and DCs were isolated from peritoneal lavage fluid of endometriosis model mice (EMs), a chronic low-grade inflammatory microenvironment. Co-culture experiments revealed that a single dose of exogenous rhIL-37 induced functional maturation of DCs. This effect was mediated through the cell membrane receptor pathway, in which rhIL-37 inhibited IL-4 production in DCs, thereby upregulating the Th1/Th2 cell ratio. This effectively reversed the tolerogenic phenotype of DCs in EMs and suppressed the progression of ectopic lesions. By contrast, another study (1) utilized an LPS-induced acute endotoxin shock model in mice-a high-intensity pro-inflammatory microenvironment-with IL-37tg mice as the research vehicle, from which splenic DCs were isolated and examined. The results showed that sustained endogenous overexpression of IL-37b significantly reduced the proportion of activated DCs and inhibited their maturation. In this system, IL-37b suppressed excessive DC activation and blocked the inflammatory cascade through intracellular and nuclear transcriptional regulation. Importantly, these two seemingly divergent findings are not mutually exclusive. Rather, they reflect differences in experimental models, IL-37 administration routes, cellular microenvironments, inflammatory backgrounds and cell sources. IL-37 functions as a bidirectional regulatory molecule whose activity is dictated by the local microenvironment: It corrects immune tolerance by promoting DC functional maturation, while simultaneously curbing excessive inflammation by suppressing DC pro-inflammatory responses.

Although endometriosis is a benign disease, it shares several hallmarks of malignancy, including migration, adhesion, invasion, proliferation and angiogenesis. Treatment with rhIL-37 or transfection with pIL-37 significantly inhibited the adhesion, migration, invasion, proliferation and angiogenesis of human or murine endometrial stromal cells by suppressing proliferating cell nuclear antigen, phosphorylated (p)-Akt, p-Erk1/2, MMP9 and VEGF expression (36,37); by contrast, IL-37 knockout enhanced these processes (36,37).

*In vivo* studies have further corroborated these findings. In a mouse model of endometriosis established by suturing uterine tissue fragments to the abdominal wall, intraperitoneal administration of rhIL-37 significantly reduced the number, weight and volume of ectopic lesions (37). Our research team developed an alternative mouse model by injecting uterine tissue fragments into the abdominal cavity; this model more accurately simulates the spontaneous adhesion, migration, invasion, lesion formation and growth of endometrial fragments refluxed into the peritoneal cavity. In this model, intraperitoneal administration of rhIL-37 similarly resulted in a marked reduction in the number, weight and volume of ectopic lesions (36), indicating that rhIL-37 treatment effectively inhibits the development of ectopic lesions.

**Figure 2:**
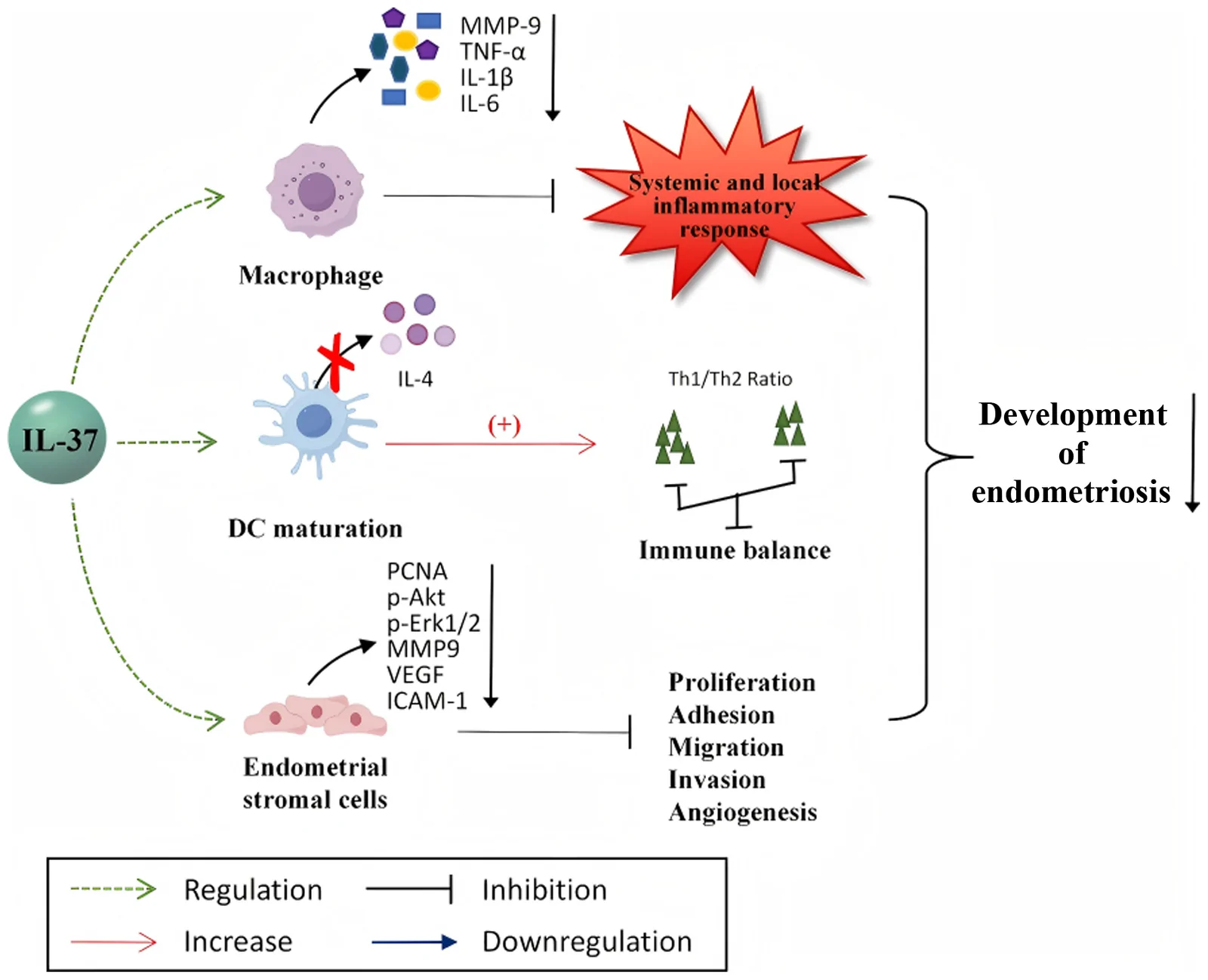
Role of IL-37 in endometriosis. Administration of rhIL-37 into the peritoneal cavity of endometriosis model mice, or *in vitro* treatment of uterine tissue explants, ectopic lesions, as well as human and mouse endometrial stromal cells, significantly reduces the expression levels of inflammatory factors in these tissues, thereby suppressing both systemic and local inflammatory responses. IL-37 regulates DCs, inhibits their production of IL-4, promotes DC maturation, increases the Th1/Th2 cell ratio and restores immune balance in the body. Furthermore, treatment with rhIL-37 or overexpression of IL-37 in human and mouse endometrial stromal cells clearly demonstrates that IL-37 inhibits cell proliferation, adhesion, migration, invasion and angiogenesis by down-regulating the expression of PCNA, p-Akt, p-Erk1/2, MMP9 and VEGF. Collectively, these findings indicate that IL-37 significantly suppresses the progression of endometriosis. DCs, dendritic cells; rhIL-37, recombinant human interleukin-37; Th1, type 1 T-helper cell; ICAM, intercellular adhesion molecule; PCNA, proliferating cell nuclear antigen; p-Erk1/2, phosphorylated extracellular signal-regulated kinase 1/2; MMP9, matrix metalloproteinase 9; VEGF, vascular endothelial growth factor.

Collectively, these studies demonstrate that IL-37 not only suppresses aberrant inflammatory mediator expression, mitigates excessive inflammatory responses and restores immune balance, and inhibits ectopic lesion growth in endometriosis models. Elevated expression of IL-37 in the peritoneal fluid, serum and ectopic lesion tissues of patients with endometriosis may serve as a potential biomarker for disease diagnosis. These findings provide experimental evidence supporting IL-37 as a promising candidate for the development of novel therapeutics for endometriosis (Fig. 2).

The role of IL-37 in gynecological and obstetric diseases is summarized in Table II.

### Role of IL-37 in adenomyosis

Adenomyosis is a gynecological condition characterized by the presence of endometrial tissue within the myometrium (54). This ectopic endometrial tissue, composed of glands and stroma, disrupts normal uterine smooth muscle contractility and peristalsis, leading to various adverse clinical outcomes (55). These effects are attributed to altered expression of pro-inflammatory and pro-angiogenic factors, which create a permissive microenvironment for endometrial tissue invasion into the myometrium (56). Studies have shown that numerous macrophages and lymphocytes accumulate in the eutopic endometrium of patients with adenomyosis (56,57). The inflammatory cytokines (such as IL-6, IL-18, IL-1β and TNF-α) and angiogenic factors (including MMP2, MMP9 and VEGF) secreted by these cells promote the invasion, proliferation and angiogenesis of adenomyotic lesions (57).

Research has demonstrated that although IL-37 is constitutively expressed in normal endometrium (9), its expression is significantly reduced in both the eutopic and ectopic endometrium of patients with adenomyosis (54,58). A similar phenomenon has been observed in hepatocellular carcinoma (HCC), where IL-37 is predominantly expressed in normal liver tissues and peritumoral liver tissues, but it is markedly reduced in tumor tissues (59). Transduction of Hep3B cells, an IL-37-negative human HCC cell line, with a lentiviral vector containing human IL-37 (Hep3B/LV-IL37) demonstrated that IL-37 overexpression recruits NK cells into tumor tissues and inhibits tumor growth (59). Another study suggested that IL-37 induces autophagy in HCC cells by inhibiting the PI3K/AKT/mTOR pathway, a mechanism that plays a crucial role in tumor suppression (60). Based on these findings, it may be speculated that IL-37 downregulation in the endometrium of patients with adenomyosis may impair local immune surveillance, thereby allowing the endometrial tissue to acquire invasive capabilities.

PTEN is a tumor suppressor that plays a critical role in modulating the PI3K-AKT-mTOR signaling pathway by regulating cellular levels of phosphatidylinositol (3,4,5)-trisphosphate (61-63). PTEN mRNA and protein expression are significantly reduced in the endometrial tissue of patients with adenomyosis, potentially due to regulation by microRNA (miR)-17 (64). MiR-17 expression is markedly elevated in the endometrial tissue of these patients and promotes adenomyosis development and progression by targeting and downregulating PTEN (64). Treatment with anti-miR-17 oligonucleotides inhibits miR-17 expression, thereby significantly upregulating PTEN expression (64). Furthermore, IL-37 has been shown to upregulate PTEN (4).

Based on the above findings, it may be proposed that anti-miR-17 oligonucleotides could be delivered to endometrial cells of patients with adenomyosis to upregulate PTEN expression. Subsequently, a viral vector encoding rhIL-37 cDNA could be introduced to further increase IL-37 expression and potentiate PTEN signaling. It may be speculated that this reconstituted anti-miR-17/IL-37/PTEN axis may exert therapeutic benefits by promoting local anti-inflammatory and anti-angiogenic responses, and normalizing cellular behavior in patients with adenomyosis. However, further experimental validation is required to confirm this hypothesis.

### Role of IL-37 in polyendocrine metabolic ovarian syndrome (PMOS)

**Figure 3:**
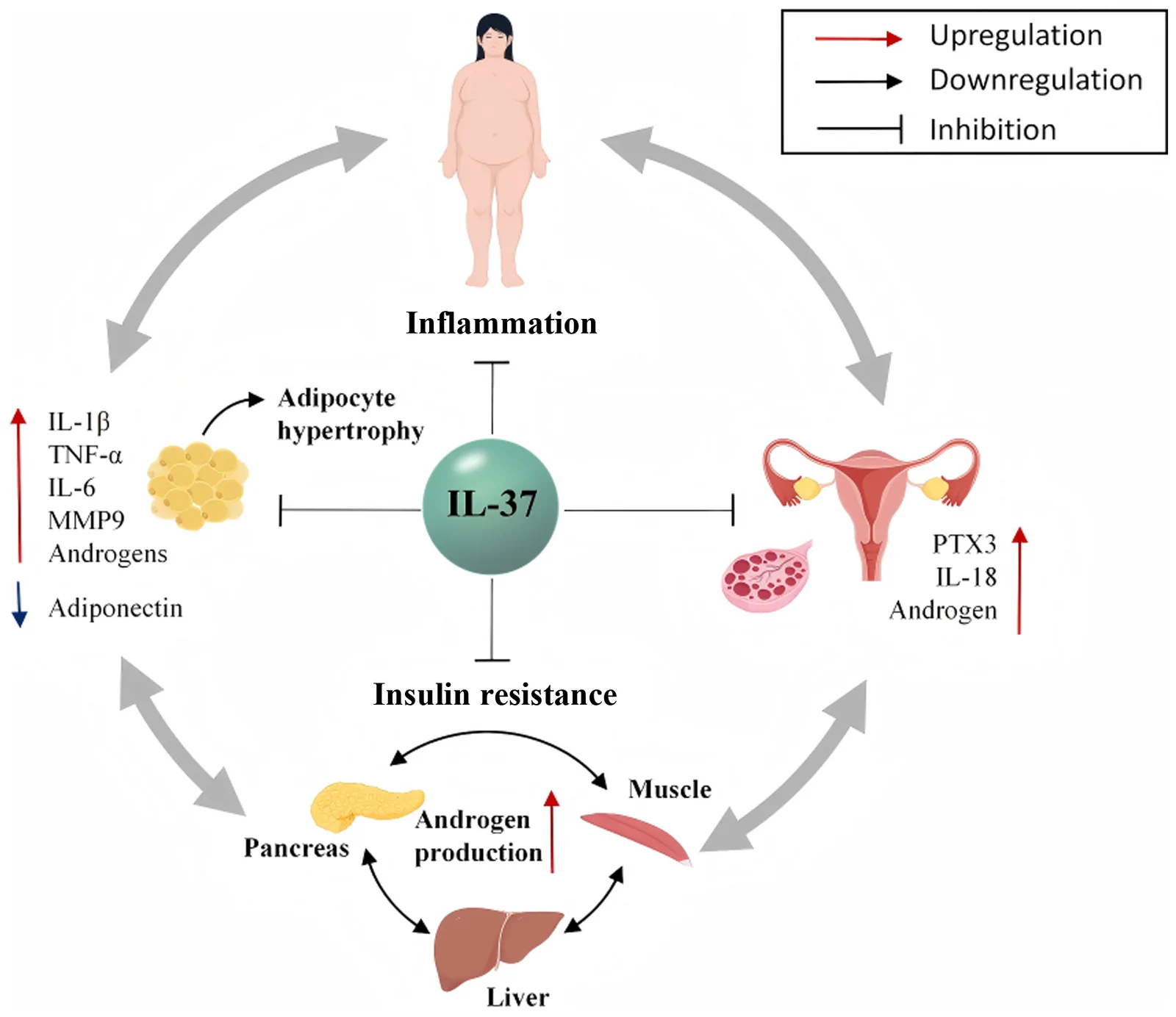
Role of IL-37 in metabolic disorders caused by PMOS. Low-grade chronic inflammation in the body stimulates adipocyte hypertrophy and macrophage infiltration, increases the release of inflammatory cytokines and androgens, and reduces adiponectin levels. Under these conditions, adipocytes become unable to store lipids, leading to lipid overflow and accumulation in other tissues, including the pancreas, liver and skeletal muscle. This results in tissue-specific insulin resistance, elevated androgen levels and inter-tissue crosstalk. Insulin resistance and hyperandrogenism subsequently interfere with normal uterine and ovarian function, leading to abnormal follicular development and ovulatory dysfunction, while also increasing the risk of endometrial hyperplasia and endometrial cancer. Together, these events-inflammation, adipocyte hypertrophy, insulin resistance, hyperandrogenism and ovulatory disorders-form a vicious cycle in PMOS patients. As an anti-inflammatory protein, IL-37 can inhibit local and systemic chronic inflammation, counteract adipocyte differentiation, enhance insulin sensitivity, improve the inflammatory microenvironment of the uterus and ovaries and thereby help ameliorate ovulatory dysfunction. IL-37, interleukin-37; PMOS, polyendocrine metabolic ovarian syndrome; PTX3, pentraxin 3.

PMOS, formerly known as polycystic ovary syndrome (PCOS), is a common reproductive and endocrine-metabolic disorder among women of reproductive age (65). The global incidence of PMOS is reported to range from 2 to 26%, underscoring the need for adequate clinical attention (66). PMOS is typically characterized by chronic persistent ovulatory dysfunction, abnormally elevated serum androgen levels and polycystic ovarian morphology, and serves as a leading cause of female reproductive infertility (67). In addition, PMOS is associated with metabolic abnormalities, including obesity, insulin resistance and impaired glucose tolerance, which increase the risk of type 2 diabetes mellitus, potential vascular diseases and endometrial adenocarcinoma, thereby significantly compromising the physical and mental health of affected women (67). Although the pathogenesis of PMOS remains incompletely understood, low-grade chronic inflammation (LGCI) has emerged as a key underlying mechanism (68,69) (Fig. 3).

PMOS-associated comorbidities are closely linked to alterations in immune function. Women with PMOS exhibit elevated levels of various immune cells in peripheral blood, including leukocytes, monocytes, lymphocytes, neutrophils and NK cells. Circulating levels of inflammatory cytokines such as TNF-α, IL-6, IL-18 and C-reactive protein (CRP) are also elevated. Among these, IL-6 can promote the activation of Th17 cells and the secretion of the inflammatory cytokine IL-17. TNF-α can directly enhance IL-6 expression and contributes to the initiation of inflammatory features in PMOS (70,71).

LGCI in patients with PMOS stimulates adipocytes and promotes the infiltration of activated immune cells into adipose tissue. Adipose tissue macrophages exhibit increased release of pro-inflammatory cytokines (TNF-α, IL-6), and dysfunctional adipocytes display an altered adipokine profile characterized by an increased leptin/adiponectin ratio. This imbalance inhibits adipocyte glucose uptake and reduces both local and peripheral insulin sensitivity (68). Concurrently, the expression and activity of aldo-keto reductase 1C3 in adipose tissue are elevated, leading to the conversion of androstenedione to testosterone and thereby increasing local androgen levels (65). Excessive androgens promote adipocyte hypertrophy and insulin resistance (65,68). Hyperandrogenism also disrupts lipid synthesis and catabolism in women with PMOS (65,68). Furthermore, in the context of hyperandrogenism and insulin resistance, dysregulated lipolysis impairs adequate lipid storage in adipocytes, resulting in ectopic lipid accumulation in the pancreas, liver and skeletal muscle. This creates a vicious cycle of tissue-specific insulin resistance, increased androgen secretion and pathological inter-organ communication (65).

As a multifunctional cytokine, IL-37 forms receptor/ligand complexes in the extracellular space via IL-18Rα/IL-1R8. The extracellular signals transmitted by this complex can inhibit the activity of pro-inflammatory cytokines such as IL-6, TNF-α, IL-1 and IL-17 (32,72,73), and suppress multiple inflammation-related signaling pathways (72). In addition, the nuclear action of the IL-37/Smad3 complex directly represses the transcription of pro-inflammatory genes (72). Collectively, these findings indicate that IL-37 can inhibit both systemic and local inflammatory responses and to modulate metabolic processes associated with PMOS. In adipose tissue culture, IL-37 significantly reduced the spontaneous secretion of IL-6, IL-1β, TNF-α and C-X-C motif chemokine ligand 1 (18) and attenuated excessive immune cell activation (1). RhIL-37 can also suppress adipogenesis in adipocytes by activating the AMPK signaling pathway (2). *In vivo* studies have demonstrated that IL-37tg mice fed a high-fat diet exhibited smaller adipocyte size and significantly reduced body weight compared with wild-type animals (5). Clinical data have shown that weight loss in obese patients correlates with elevated IL-37 levels (18,74). These results suggest that IL-37 can ameliorate the adipose tissue microenvironment and regulate body weight. In a study on obesity-related inflammation, higher IL-37 mRNA expression in adipose tissue was associated with improved insulin sensitivity (5). Following 22 weeks of high-fat diet feeding, wild-type mice treated with rhIL-37 for two weeks exhibited reduced plasma insulin concentration and improved glucose tolerance, and increased pancreatic insulin content (18). The anti-inflammatory effects of rhIL-37 can ameliorate established metabolic disturbances in PMOS (75), and similar results were obtained in IL-37tg mice (5). These findings indicate that IL-37 may represent a potential therapeutic target for metabolic disorders in PMOS.

Physiological levels of inflammation are necessary for follicular development and ovulation; however, low-grade chronic inflammation can lead to oocyte defects and ovulatory dysfunction (76). Studies have reported elevated IL-18 levels in the follicular fluid of patients with PMOS. Pentraxin 3 (a member of the CRP superfamily) is significantly increased in granulosa cells of patients with PMOS compared with non-PMOS controls (77). Extensive infiltration of activated immune cells into the granulosa cell layer and theca layer of the ovaries has been observed. This inflammatory factor secreted by these cells mediates follicular atresia or apoptosis, ultimately resulting in ovulatory dysfunction (78,79). Although IL-37 expression is known to be low in patients with PMOS, exogenous IL-37 can block M1 macrophage polarization by inhibiting the Notch1 and NF-κB pathways (80). Furthermore, IL-37 significantly inhibits the biological activity of IL-18 and suppresses various abnormally expressed inflammatory factors through binding to the IL-18BP/IL-18Rβ complex (4), thereby attenuating inflammation and improving the ovarian microenvironment as well as ovulatory function.

Hyperandrogenism is the most consistently observed feature in women with PMOS (81). Biochemical hyperandrogenism is defined as a free androgen index (FAI) value exceeding 4.5, and patients with PMOS exhibit significantly higher FAI values compared with controls (82). It has been reported that androgen levels in patients with PMOS are markedly elevated irrespective of body mass index (82). *In vitro* cell culture experiments have shown PMOS theca cells maintained equivalent testosterone after 22-38 passages compared with primary cultures of PMOS theca cells, suggesting that hyperandrogenism represents a stable phenotypic feature of PMOS theca cells (65,83). Enzymes involved in androgen biosynthesis, including cytochrome P450 family 17 subfamily A member 1 and 3β-hydroxysteroid dehydrogenase type 2, are overexpressed in ovarian theca cells (83). The effects of androgens are mediated by androgen receptors (ARs) and play a crucial role in the progression of PMOS (84). Previous reports have indicated that Smad3 is a critical component in the androgen signaling pathway, physically interacting with AR and serving as an auxiliary regulator for AR (85). It has been shown that the binding of AR to intron 3 of the AR gene requires the involvement of Smad3, thereby promoting AR mRNA expression. Knockout of SMAD3 leads to reduced AR binding to chromatin as well as decreased expression of ARs and their target genes (86). Another study reported that IL-37 acts as a negative regulator of the Smad pathway in tumor cells, competing with Smad2 or Smad4 for binding to Smad3, thereby inhibiting tumor cell proliferation, invasion and metastasis (31). It may be hypothesized that in patients with PMOS, IL-37 binding to Smad3 may alter its three-dimensional conformation in a manner that prevents Smad3-AR interaction or binding of the Smad3/AR complex to intron 3 of the AR gene, suggesting IL-37 could serve as a potential therapeutic strategy for hyperandrogenism. This hypothesis, however, requires experimental validation. Although no reports to date have directly demonstrated that IL-37 ameliorates hyperandrogenism, available evidence indicates that FAI is positively correlated with pro-inflammatory factors such as TNF-α, CRP and α1-acid glycoprotein, and negatively correlated with anti-inflammatory factors including IL-27, IL-25 and IL-37 (82). Collectively, these findings suggest that IL-37 plays a beneficial therapeutic role in ameliorating the symptoms of hyperandrogenism.

## 3. IL-37 and obstetric diseases

### IL-37 in gestational diabetes mellitus (GDM)

GDM is defined as hyperglycemia that develops during gestation in women without a prior history of diabetes (87). More specifically, GDM typically manifests between 24 and 28 weeks of pregnancy, when insulin resistance leads to blood glucose levels that are higher than normal (88). GDM is a metabolic disorder accompanied by a chronic inflammatory response (89). Studies have shown that the mRNA expression levels of IL-6, IL-1β, TNF and NF-κB in the placenta of patients with GDM are significantly increased, whereas IL-37 levels in serum, placental villi, umbilical artery and umbilical vein are markedly decreased (89,90). This reduction in IL-37 levels weakens the inflammatory protective effects of the chorionic villi and umbilical cord, thereby further exacerbating the placental inflammatory response (89,90).

Additionally, adipose tissue-derived leptin impairs insulin-dependent glucose transport into adipocytes (91,92), thereby accelerating the onset of insulin resistance. NF-κB is a signaling molecule that plays a critical role in immune and inflammatory responses, promoting adipocyte inflammation and impairing insulin-mediated glucose uptake (89,93). As previously noted, elevated IL-37 levels exert anti-inflammatory effects and enhance insulin sensitivity (5). IL-37 suppresses inflammation by inhibiting the NF-κB pathway and thus plays an important role in alleviating insulin resistance in women with GDM (89).

It has been demonstrated that miR-657, a non-coding RNA, significantly promotes the proliferation of THP-1 macrophage-like cells upon phorbol 12-myristate 13-acetate (PMA) stimulation and enhances inflammatory responses and tumorigenesis (94). Furthermore, a negative correlation between miR-657 and IL-37 expression levels has been observed in placental tissue. Compared with normal pregnancy, miR-657 expression is elevated in placental tissue cells of women with GDM (94). MiR657 targets nucleotides 59-65 within the 3'untranslated region of the IL-37 mRNA transcript, thereby repressing IL-37 protein expression. Notably, transfection of rhIL-37 into THP-1 cells reversed the effect of miR-657 (94). These findings suggest that exogenous rhIL-37 therapy can effectively counteract the effects of miR-657 at the cellular level. A recent report further indicated that changes in the interleukin profile, particularly decreased IL-37 levels, were detected in the serum of postpartum women with GDM (95). Therefore, IL-37 may also serve as a potential biomarker of immune dysfunction in the early postpartum period following GDM.

### IL-37 in pre-eclampsia

Pre-eclampsia is characterized by the new onset of hypertension and proteinuria after 20 weeks of gestation, often accompanied by dysfunction of the cardiovascular, neurological, visual or renal systems and affects ~4-5% of pregnancies worldwide (96). An imbalance between regulatory receptors or anti-inflammatory factors and pro-inflammatory cytokines, driven by chronic inflammation and oxidative stress, is considered a primary mechanism underlying hypertension in pre-eclampsia. This condition can lead to fetal nutrient deprivation, hypoxia and growth restriction (97,98).

Studies have reported that in women with pre-eclampsia, the placenta releases large quantities of both pro-inflammatory and anti-inflammatory mediators (97). Compared with normal pregnant women, IL-37 production in the placenta of women with pre-eclampsia is increased by >5-fold (97). Concurrently, patients with pre-eclampsia also exhibit higher serum IL-37 concentrations than healthy pregnant women (98,99). *In vitro* experiments have demonstrated that cells cultured under 1% oxygen express twice as much IL-37 as those cultured under 20% oxygen (97). These findings indicate that IL-37 expression is induced by both hypoxia and inflammation.

Despite the complex and elusive pathogenesis of pre-eclampsia, all studies evaluating its risk factors have provided highly consistent evidence of a clear association between insulin resistance and the disease (100). Relieving insulin resistance may reduce the incidence of pregnancy-induced hypertension (101). It is well established that elevated IL-37 serves as a feedback mechanism that prevents excessive tissue damage caused by inflammatory responses. Furthermore, IL-37 has been shown to improve insulin sensitivity (5,89). Collectively, these findings suggest that IL-37 may play a critical role in the prevention and treatment of pre-eclampsia.

### IL-37 in spontaneous preterm birth (sPTB)

Normal childbirth typically occurs between 37 and 42 weeks of gestation. Human parturition is an inflammatory process that leads to uterine contraction, cervical ripening and membrane activation/rupture, ultimately resulting in successful fetal delivery (102). Cytokines play a key role in the initiation and regulation of this process (103). sPTB, defined as delivery occurring before 37 weeks of gestation, is considered a classic inflammatory disease (104,105). In cases of premature rupture of fetal membranes, total leukocyte chemotactic activity appears to be greater than that observed in normal rupture (105). Numerous macrophages and neutrophils infiltrate the fetal membranes and contribute to the stimulation of inflammatory mediators associated with membrane activation (103,105). NF-κB, activated by pro-inflammatory cytokines, plays an important role in stimulating uterine contractions and cervical ripening, as it is required for prostaglandin synthesis and the regulation of matrix metalloproteinase (MMP) expression. Premature or pathological activation of NF-κB may subsequently lead to spontaneous preterm birth (103,104).

IL-37, a natural suppressor of inflammatory and immune responses, exhibits decreased mRNA and protein levels in both human plasma and fetal membranes in an sPTB group, suggesting its potential involvement in the pathogenesis of sPTB (106). Furthermore, *in vitro* studies have demonstrated that IL-37 inhibits the production of IL-1β, IL-6 and TNF-α in the human amniotic epithelial cell line WISH, indicating that IL-37 may help maintain local immune balance by limiting excessive inflammation at the maternal-fetal interface (11,106). In IL-37-treated WISH cells, MMP9 expression was significantly downregulated (11,106), which may help prevent the degradation of the fetal membrane extracellular matrix and the production of prostaglandins associated with preterm birth. Additionally, in the presence of lipopolysaccharide (LPS), the expression of p-NF-κB and p-STAT3 in IL-37-treated WISH cells was significantly lower than that in the control group (106). Furthermore, cell apoptosis is one of the factors contributing to preterm birth; knockout of IL-37 significantly promoted LPS-induced apoptosis in WISH cells (11). Based on current experimental findings, it is hypothesized that IL-37 may play a protective role in preventing premature or pathological uterine contractions, cervical ripening and membrane rupture by inhibiting excessive inflammation, MMP9 expression and NF-κB activation.

## 4. IL-37 in systemic diseases affecting the reproductive system

### IL-37 in HIV

Acquired immunodeficiency syndrome is a sexually transmitted disease caused by the human immunodeficiency virus (HIV), a lentivirus belonging to the Retroviridae family (107,108). Based on genetic and antigenic variations, HIV is classified into two types: HIV-1 and HIV-2. HIV-1 is the more virulent type and is responsible for the global pandemic (108,109). Compared with uninfected individuals, HIV-1-infected individuals exhibit significantly elevated levels of IL-37 mRNA levels (11,110). Further study has shown that IL-37 protein levels are also elevated in the serum of HIV patients (23). This elevated IL-37 expression may play a regulatory role in HIV-1 infection by limiting inflammation and inhibiting HIV replication in human T cells (23). In addition, peripheral blood mononuclear cells from HIV-infected patients lack expression of the IL-37b isoform, and expression of the IL-37 receptor IL-1R8 is reduced in immune cells (including monocytes, B cells and natural killer cells); Circulating sIL-1R8 concentrations are markedly elevated in patients. *In vitro* assays have demonstrated that exogenous sIL-1R8 substantially blunts the IL-37-mediated suppression of TNF-α production in LPS-stimulated THP-1 cells, indicating impaired function of the IL-37b/IL-1R8 axis in HIV-infected individuals (23). Targeting the IL-37b/IL-1R8 axis may inhibit both inflammatory responses and viral replication of HIV, which would undoubtedly be beneficial for patients with HIV.

### IL-37 in Behcet's disease (BD)

BD is a chronic inflammatory disorder affecting multiple organs. It is primarily characterized by recurrent oral mucosal ulcers, genital ulcers, ophthalmitis and other skin lesions; its etiology has remained elusive (111). The expression levels of IL-37 mRNA and protein are significantly reduced in peripheral blood mononuclear cells of patients with active BD (12). Experiments have demonstrated that upregulation of thymic stromal lymphopoietin (TSLP) and IL-33 expression in patients with BD may contribute to cutaneous inflammation; however, rhIL-37 can inhibit TSLP and IL-33 expression levels (112). Treatment of monocyte-derived dendritic cells with rhIL-37 not only reduces IL-1β, IL-6 and TNF-α but also upregulates IL-27 expression (113). Furthermore, rhIL-37 suppresses the generation of reactive oxygen species, decreases the activation of p38MAPK, ERK1/2 and JNK, and suppresses Th17 and Th1 responses by modulating dendritic cell function (113). Collectively, these findings suggest that rhIL-37 treatment may improve the clinical symptoms of BD.

## 5. Role of IL-37 in gynecological tumors

In addition to its anti-inflammatory and anti-metabolic disorder properties, IL-37 also exhibits anti-tumor activities. Recent studies have demonstrated progress in understanding the role of IL-37 in gynecological tumors, including breast cancer, cervical cancer, epithelial ovarian cancer and endometrial carcinoma (see Table III).

### Breast cancer

Breast cancer is one of the most common malignancies in women and a leading cause of cancer-related death (114). However, the precise immunopathological mechanisms underlying this disease remain elusive. Previous studies have shown that when recombinant adenoviral vectors containing human IL-37 cDNA fragments were transduced into 4T1 cells (a breast cancer cell line), the resulting IL-37-expressing 4T1 cancer cells formed smaller tumors upon implantation into the mammary fat pads compared with control groups (115). Further experiments indicated that IL-37 inhibits the growth of 4T1 breast cancer cells by promoting the activation and proliferation of CD4+ T cells and modulating the tumor microenvironment (115,116).

Extensive research has demonstrated that breast cancer exhibits metastatic heterogeneity, resulting in variable prognosis and treatment response among patients. Studies have shown that estrogen receptor-positive/human epidermal growth factor receptor 2-positive (ER+/HER2+) and estrogen receptor-negative/HER2-positive (ER-/HER2+) breast cancers exhibit distinct metastatic patterns. Patients with ER-/HER2+ breast cancer had significantly higher rates of multiple metastases and lower survival rates than those with ER+/HER2+ breast cancer (117). Another study revealed that the relative expression level of IL-37 in blood samples of ER+/progesterone receptor+/HER2+ patients (non-metastatic breast cancer) was significantly higher than that in HER2+ patients (118). Furthermore, compared with healthy controls, both patients with metastatic and non-metastatic breast cancer had significantly lower CD8+ T-cell counts in blood samples (118,119). These findings suggest that measuring IL-37 levels and CD8+ T-cell counts may help elucidate their relationship with breast cancer metastasis and inform the development of novel treatment strategies.

MDA-MB-231 and MCF-7 are two additional human breast cancer cell lines. The former is metastatic and exhibits low IL-37 expression, whereas the latter is non-metastatic and shows higher IL-37 expression (31). Low IL-37 expression in tumors is significantly associated with poor prognosis in cancer patients. MDA-MB-231 cells overexpressing IL-37b via transgenic technology exhibited significantly decreased expression of inflammatory factors, integrins and MMP-9, thereby suppressing tumor cell invasiveness and metastasis. Conversely, IL-37b knockdown in MCF-7 cells enhanced these malignant phenotypes (31). Therefore, IL-37 may represent a potential therapeutic target for tumors, particularly metastatic tumors.

### Cervical cancer (CC)

CC is the fourth most frequent cancer in women worldwide, second only to breast cancer (120,121). Human papillomavirus infection and chronic inflammation are considered major pathogenic factors in the development of CC (122). IL-37 expression is decreased in CC and this reduction is associated with poor prognosis of the disease.

Previous studies have shown that IL-37 inhibits the proliferation and invasion of CC cells (HeLa and C33A cells) by suppressing STAT3 expression and STAT3 phosphorylation (123). Further research suggested that activation of runt-related transcription factor 2 (RUNX2) in cancer cells is associated with tumorigenesis, metastasis and invasion (124). In Siha and C33A CC cells transfected with IL-37, cancer cell invasion was significantly inhibited and RUNX2 expression (at both the mRNA and protein levels) was markedly reduced, indicating that the inhibitory effect of IL-37 on the invasion of these two cell lines is mediated through downregulation of RUNX2 expression (124).

Cell apoptosis is considered a tumor-suppressive mechanism. Bim, a pro-apoptotic molecule consisting of 198 amino acids, plays a key role in this process (120). IL-37 has been shown to upregulate Bim expression and induce apoptosis in CC cell lines HeLa and C33A (120). Additionally, a recent study demonstrated that IL-37 can suppress tumor immune evasion by downregulating CD47 expression in CC cells, thereby enhancing macrophage-mediated phagocytosis of tumor cells (125).

In summary, IL-37 inhibits the progression of CC by regulating the expression of factors involved in cancer cell proliferation, invasion, apoptosis and immune escape.

### Epithelial ovarian cancer

Epithelial ovarian cancer is an aggressive malignancy, and one of the primary factors contributing to its high mortality rate is the advanced stage of the disease at the time of diagnosis (126). Therefore, exploring novel approaches for molecular diagnosis and molecular therapy is particularly important for early detection and treatment. Huo *et al* (127) reported that serum IL-37 protein levels in patients with epithelial ovarian cancer were significantly higher than those in healthy individuals, and patients with International Federation of Gynaecology and Obstetrics (FIGO) stage III/IV epithelial ovarian cancer exhibited markedly higher IL-37 levels than those with FIGO stage I/II disease. Elevated serum IL-37 levels in patients with ovarian cancer were associated with adverse survival outcomes. Furthermore, analysis revealed that IL-37 expression levels were positively correlated with tumor size, lymph node metastasis and recurrence (127). These findings suggest that IL-37 may serve as a clinically useful indicator of epithelial ovarian cancer progression and as a potential biomarker for predicting patient survival prognosis. However, to the best of our knowledge, to date, no reports have addressed the precise biological role of IL-37 in epithelial ovarian cancer.

### Endometrial carcinoma

Endometrial carcinoma is one of the most common gynecological malignancies (128). Like other tumor cells, endometrial cancer cells possess migratory and invasive properties (129). *In vitro* experiments have demonstrated that IL-37bΔ1-45 (the mature form of IL-37b) significantly inhibits the malignant phenotype of endometrial cancer cells, including invasion, migration and metastasis; furthermore, *in vivo* experiments have yielded consistent results (124). MMP2 contributes to both tumor growth and metastasis. Further experiments have shown that IL-37bΔ1-45 markedly reduces MMP2 expression levels in endometrial cancer cells and suppresses their migration and invasion by targeting the Rac1/NF-κB/MMP2 signaling pathway (11,129).

## 6. Conclusions

Substantial evidence indicates that chronic inflammatory stimuli and the resulting inflammatory microenvironment may promote the development of gynecological and obstetric diseases as well as gynecological tumors. Therefore, understanding the occurrence and progression of these related diseases is crucial for developing interventions to promote women's health. IL-37 plays an important role in the diagnosis and treatment of gynecological and obstetric diseases and gynecological tumors by inhibiting the expression of pro-inflammatory cytokines, blocking excessive inflammatory responses and regulating gene transcription, cellular metabolism and cell proliferation through both intracellular and extracellular mechanisms.

For instance, endometriosis exhibits tumor-like features and IL-37 suppresses excessive inflammatory responses through multiple signaling pathways, significantly reducing the number, size and weight of ectopic lesions. In patients with PMOS, IL-37 inhibits inflammation, reduces body weight, increases circulating adiponectin levels and improves insulin resistance, glucose tolerance and ovulatory dysfunction. In spontaneous preterm birth, IL-37 plays a protective role by maintaining local immune balance, thereby helping to prevent preterm delivery or pathological uterine contractions, cervical ripening and membrane activation/rupture. In HIV-infected individuals, IL-37 inhibits viral replication. In several gynecological tumors, including breast cancer, CC and endometrial carcinoma, IL-37 suppresses tumor progression by regulating the biological functions of cancer cells, inhibiting proliferation, migration, invasion and angiogenesis.

Compared with healthy individuals, the expression levels of IL-37 are significantly altered in patients with gynecological diseases and tumors. This characteristic may serve as a valuable biomarker for disease diagnosis and prognostic prediction.

In recent years, research on IL-37 has garnered increasing attention in this field. After a systematic literature search, no existing evidence was found regarding the expression or regulation of other IL-37 subtypes in gynecological diseases. Owing to limitations in current detection technologies, almost all clinical specimens obtained from gynecological patients-including serum, peritoneal fluid, eutopic/ectopic endometrium and cell culture supernatants-are only capable of detecting total IL-37. Similarly, almost all mouse specimens, derived from disease models injected with rhIL-37b protein, rhIL-37 transgenic mice and mouse tissues or cells treated with rhIL-37b protein, have been analyzed for IL-37b exclusively. These findings collectively demonstrate the roles of IL-37 and IL-37b in various disease contexts. It is well established that IL-37b is the most structurally complete and biologically active isoform among all IL-37 subtypes. Although the detection of total IL-37 can indirectly reflect the levels and functions of IL-37b, the potential contributions of other IL-37 subtypes cannot be excluded. To specifically identify distinct IL-37 subtypes, the use of subtype-specific primers, subtype-specific antibodies and subtype-specific ELISA kits would be required. With continued advances in detection methodologies, this limitation is expected to be overcome in the future. Future efforts should focus on expanding the scope of clinical studies, further validating the clinical utility of IL-37 as both a biomarker and a therapeutic target and providing robust evidence to support its development as a novel therapeutic agent.

## Figures and Tables

**Table I:** Expression levels of IL-37 in gynecological and obstetric diseases as well as gynecological tumors.

IL-37 expression	Diseases
Elevated	• Endometriosis • Pre-eclampsia • HIV • Non-metastatic breast cancer • Epithelial ovarian cancer
Decreased	• Adenomyosis • Polyendocrine metabolic ovarian syndrome • Gestational diabetes mellitus • Spontaneous preterm birth • Behcet's disease • Metastatic breast cancer • Cervical cancer • Endometrial carcinoma

IL-37, interleukin-37.

**Table II:** Roles of IL-37 in gynecological and obstetric diseases.

Disease	Role of IL-37	Pathway	(Refs.)
Endometriosis	IL-37 reduces the levels of IL-1β, TNF-α, IL-6, VEGF, soluble ICAM-1 and MMP-2/9, regulates immune balance and inhibits the growth of ectopic lesions.	Through inactivation of Wnt/β-catenin, p38 MAPK, Akt, ERK1/2-MAPK, JNK and NF-κB pathways.	(36,37,52)
Adenomyosis	Downregulation of IL-37 may inhibit the expression of PTEN, leading to the occurrence of adenomyosis. Further research is needed on this result.	Downregulation of IL-37 may activate the PI3K-AKT-mTOR signaling pathway.	(4,58,60,64)
Polyendocrine metabolic ovarian syndrome	Suppression of inflammation; weight loss; increase of adiponectin circulating levels; improvement of ovulation disorders, insulin resistance and glucose tolerance; decreased lipogenesis of adipocytes.	Inhibition of inflammation via IL-37-IL-18Rα-IL-1R8, IL-37-IL-18BP-IL-18Rβ, IL-37/Smad3 pathways. Reduction of plasma insulin levels and islet mass by inhibiting mTOR and activating AMPK.	(4,5,18,72-74)
HIV	Improvement of inflammation. Inhibition of HIV replication.	Relates to impaired function of IL-37/IL-1R8 axis	(11,23,107,110)
Behcet's disease	IL-6, IL-1β, TNF-α, ROS ↓ IL-27 ↑ Th17, Th1 responses ↓ Inhibition of TSLP-skin synthesis; Improvement of the clinical symptoms.	Reduction of the activation of ERK1/2, JNK and p38MAPK	(112,113)
Gestational diabetes mellitus	Anti-inflammation and enhancement of insulin sensitivity. rhIL-37 is capable of preventing against the effect of miR-657 in THP-1 cells of transfected IL-37.	By inhibiting the NF-κB pathway; Unclear	(5,89,94)
Pre-eclampsia	Preventing excessive damage mediated by inflammatory response.		
	IL-37 may reduce the risk of gestational hypertension by reducing insulin resistance.	Unclear.	(5,97)
Spontaneous preterm birth	Repression of the levels of inflammatory factors, such as TNF-α, IL-1β and IL-6. Suppression of excessive inflammation, ECM remodeling and apoptosis.	Repression of the NF-κB and IL-6/STAT3 pathways.	(11,106)

IL-1β, interleukin-1β; TNF-α, tumor necrosis factor α; IL-18Rα, interleukin-18 receptor α chain; IL-18BP, IL-18 binding protein; IL-1R8, interleukin-1 receptor 8; Smad3, SMAD family member 3; Th1, type 1 T-helper cell; TSLP, thymic stromal lymphopoietin; rhIL-37, recombinant human IL-37; miR, microRNA; VEGF, vascular endothelial growth factor; ICAM-1, intercellular adhesion molecule 1; MMP-2/9, matrix metalloproteinase 2/9; PTEN, phosphatase and tensin homolog; ROS, reactive oxygen species; ECM, extracellular matrix; PI3K, phosphoinositide 3-kinase; AKT, protein kinase B; mTOR, mammalian target of rapamycin; AMPK, AMP-activated protein kinase; Wnt, Wingless-related integration site; p38 MAPK, p38 mitogen-activated protein kinase; ERK1/2, extracellular signal-regulated kinase 1/2; JNK, c-Jun N-terminal kinase; NF-κB, nuclear factor κB; STAT3, signal transducer and activator of transcription 3.

**Table III:** Roles of IL-37 in gynecological tumors.

Disease	Role of IL-37	Pathway	(Refs.)
Breast cancer	Inhibition of the proliferation of 4T1 breast cancer cells. Reduction of the expression of inflammatory factors, integrin and MMP-9 in MDA-MB-231 cells and inhibition of their invasion and metastasis.	Suppression of the activation of ERK, p38 MAPK, JNK, PI3K, NF-κB and STAT3 pathways.	(31,115,118,119)
Cervical cancer	Inhibition of the proliferation and invasion of Hela and C33A cells. Downregulation of RUNX2 expression. Upregulation of Bim expression and induction of apoptosis in HeLa and C33A cells. Suppression of tumor immune evasion and enhancement of macrophage phagocytosis by downregulating the expression of CD47.	Suppression of the expression of STAT3 and the phosphorylation of STAT3.	(120,123-125)
Epithelial ovarian cancer	The expression level of IL-37 is positively correlated with tumor size, lymph node metastasis and positive recurrence, and can serve as a potential marker for clinical epithelial ovarian cancer diagnosis and prognosis.	Unclear.	(127)
Endometrial carcinoma	Reduction of the expression level of MMP-2. Suppression of the migration and invasion of endometrial cancer cells.	By targeting the Rac1/NF-κB/MMP-2 signaling pathway.	(11,129)

IL-37, interleukin-37; MMP-9, matrix metalloproteinase 9; ERK, extracellular signal-regulated kinase; p38 MAPK, p38 mitogen-activated protein kinase; JNK, c-Jun N-terminal kinase; PI3K, phosphoinositide 3-kinase; NF-κB, nuclear factor κB; STAT3, signal transducer and activator of transcription 3; RUNX2, runt-related transcription factor 2; CD47, cluster of differentiation 47; Rac1, Ras-related C3 botulinum toxin substrate 1.

## Data Availability

Not applicable.
